# Does Robotic Segmentectomy Improve Long-Term Outcomes in Non-Small Cell Lung Cancer?

**DOI:** 10.1093/ejcts/ezag048

**Published:** 2026-01-28

**Authors:** Giulia Fabbri, Nabih Berjaoui, Savvas Lampridis, Alessandro Maraschi, Karen Harrison-Phipps, Tom Routledge, Andrea Dell'Amore, Akshay Patel, Andrea Bille

**Affiliations:** Department of Medicine - DIMED, University of Padua, Italy; Department of Thoracic Surgery, Guy’s and St Thomas NHS Foundation Trust, London, SE1 9RT, United Kingdom; Department of Thoracic Surgery, Azienda Ospedale-Universitaria di Padova, Via Giustiniani, 2, Padova, 35128, Italy; Department of Thoracic Surgery, Guy’s and St Thomas NHS Foundation Trust, London, SE1 9RT, United Kingdom; Department of Thoracic Surgery, Guy’s and St Thomas NHS Foundation Trust, London, SE1 9RT, United Kingdom; Department of Thoracic Surgery, Guy’s and St Thomas NHS Foundation Trust, London, SE1 9RT, United Kingdom; Department of Thoracic Surgery, UCLH Hospital, 16-18 Westmoreland Street, London, W1G 8PH, United Kingdom; Department of Thoracic Surgery, Guy’s and St Thomas NHS Foundation Trust, London, SE1 9RT, United Kingdom; Department of Thoracic Surgery, Guy’s and St Thomas NHS Foundation Trust, London, SE1 9RT, United Kingdom; Department of Medicine - DIMED, University of Padua, Italy; Department of Thoracic Surgery, Azienda Ospedale-Universitaria di Padova, Via Giustiniani, 2, Padova, 35128, Italy; Department of Thoracic Surgery, Guy’s and St Thomas NHS Foundation Trust, London, SE1 9RT, United Kingdom; Department of Thoracic Surgery, Guy’s and St Thomas NHS Foundation Trust, London, SE1 9RT, United Kingdom

**Keywords:** robotic-assisted thoracic surgery, video-assisted thoracic surgery, segmentectomy, non-small cell lung cancer, long-term outcomes, lymphadenectomy

## Abstract

**Objectives:**

Lung cancer remains the leading cause of cancer-related death globally. While lobectomy is the standard treatment for early-stage disease, trials have shown that segmentectomy offers comparable survival outcomes for small (≤2 cm) peripheral tumours. Robotic-assisted thoracic surgery (RATS) has gained popularity due to enhanced precision compared with video-assisted thoracic surgery (VATS). However, comparative outcomes of RATS vs VATS segmentectomy remain unclear. This study, therefore, aimed to compare short-term and long-term outcomes in patients undergoing VATS and RATS segmentectomy for non-small cell lung cancer (NSCLC).

**Methods:**

We retrospectively reviewed consecutive patients undergoing RATS or VATS segmentectomy for NSCLC between July 2015 and December 2021. Primary outcomes were overall survival (OS), disease-free survival (DFS), and recurrence. Secondary outcomes included complications, length of stay, length of drainage, and lymph-node stations harvested.

**Results:**

A total of 144 patients were included (RATS *n* = 86; VATS *n* = 58). Baseline characteristics were comparable across groups. RATS was associated with a significantly greater number of lymph-node stations harvested and wider tumour-free margins. Short-term outcomes, including complications, length of stay, drainage duration, conversion rates, and operative time, were similar. Long-term outcomes favoured the robotic approach, with significantly improved OS, DFS, and lower recurrence rates, although multivariable analysis showed no significant difference in hazard ratios between approaches.

**Conclusions:**

RATS segmentectomy demonstrated improved survival metrics and reduced recurrence compared with VATS while maintaining comparable perioperative outcomes. The robotic platform facilitated more extensive lymphadenectomy and wider resection margins, which may underlie the observed oncologic advantages.

## Graphical Abstract

**Figure:**
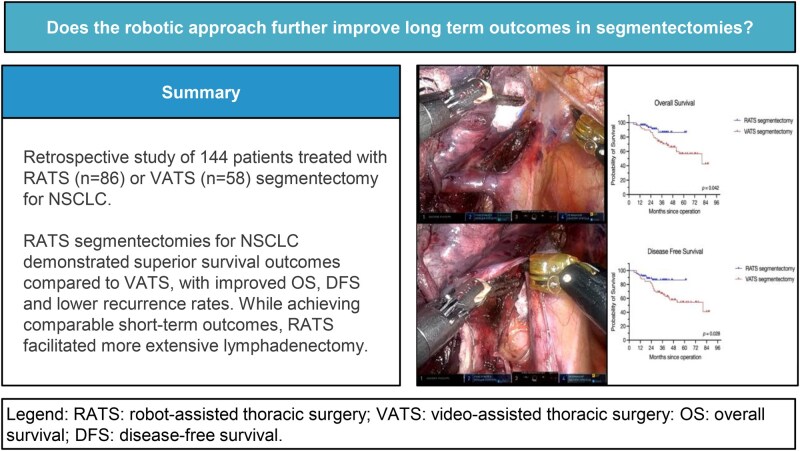

## Introduction

Lung cancer is the leading cause of cancer death worldwide.^1^ For early-stage disease, lobectomy is currently considered the gold standard treatment. The JCOG0802/WJOG4607L trial showed that segmentectomy is superior to lobectomy in terms of overall survival (OS) for peripheral tumours ≤2 cm, while having similar 5-year relapse-free survival, but this difference was not seen at the 10-year follow-up.^2^ Further to this, the CALBG 140503 trial showed that sublobar resection in patients with pN0 tumours ≤2 cm was not inferior to lobectomy with respect to disease-free survival (DFS), and had similar results in terms of OS.^3^ Consequently, segmentectomies have been increasingly adopted in surgical practice to resect small peripheral tumours. Nonetheless, segmentectomies, especially complex segmentectomies, often require a more thorough and challenging parenchymal dissection compared to lobectomies.

In recent years, robotic-assisted thoracic surgery (RATS) has been increasingly used for lung resection surgery because of its advantageous technical features, such as three-dimensional visualization and multi-wristed instrumentation that allow a more thorough and precise dissection compared to video-assisted thoracic surgery (VATS). Numerous studies have suggested that RATS lobectomy might be associated with similar or even better short-term outcomes,^4–7^ and similar long-term survival rates compared with VATS.^8–9^

However, there is a lack of evidence in current literature assessing the surgical and oncologic outcomes in VATS and RATS segmentectomies. Therefore, the aim of our study was to compare these approaches in terms of postoperative outcomes and long-term survival in patients undergoing anatomical segmentectomy for early-stage non-small-cell lung cancer (NSCLC).

## Materials and methods

### Patient selection

We retrospectively reviewed all consecutive adult patients who underwent RATS or VATS segmentectomy for NSCLC at Guy’s Hospital (Guy’s and St Thomas NHS Foundation Trust, London, United Kingdom) between July 2015 and December 2021.

Exclusion criteria included tumours measuring over 2 cm on preoperative computed tomography (CT) scan, lymph node disease at clinical staging, concurrent or previous primary cancers, small-cell lung cancer, and incomplete pathological resection.

Patients were characterized according to demographic variables, including age, sex, smoking history (never, former, and current smokers), clinical variables, namely performance status (<2 or ≥2), comorbidities, whether they received adjuvant therapy or not, forced expiratory volume in 1 s (FEV1), diffusing capacity of the lungs for carbon monoxide (DLCO), and surgical variables, such as type of segmentectomy (complex or simple), and tumour histology.

Patients operated on before the adoption of the 8th edition of the TNM classification of malignant tumours were restaged according to the 8th edition.

### Outcomes

The primary outcomes were overall survival (OS), recurrence rate, and DFS. Secondary outcomes included postoperative complication rates, length of hospital stay (LOS), length of chest drain (LOD), mortality, conversion rate, operating time, number of lymph node stations harvested, nodal upstaging rate, and resection margin free of tumour. As “margin free of tumour,” we considered the distance between the tumour and the bronchial staple line.

### Follow-up

Patients were followed up after surgery according to institutional guidelines. Follow-up visits were scheduled every 6 months for the first 2 years after surgery, then annually thereafter. At each follow-up visit, patients underwent physical examination and thoracic CT. Positron emission tomography integrated with CT was performed if recurrence was suspected based on symptoms or other imaging findings. Patients were defined as lost to follow-up when they did not return for at least 2 consecutive follow-ups and the study team was unable to reach them.

Recurrence was defined as the presence of new lesions on imaging consistent with metastatic disease, along with a biopsy confirmation, when possible. Sites and dates of the first recurrence were recorded. OS was determined as the time from surgery until death from any cause or loss to follow-up. Patients who did not die during the observation period were censored at the date of the last available follow-up. DFS was defined as the time from surgery until recurrence or death from any cause.

### Surgical technique

All surgical procedures were performed by 2 fully accredited thoracic surgeons. RATS segmentectomies were performed using the Da Vinci Xi Surgical Platform (Intuitive Surgical, Inc., Santa Clara, CA, USA) via 4 robotic ports (2 8 mm ports and 2 12 mm ports) plus an additional port for bedside assistance and specimen retrieval. CO_2_ at a pressure of 6-8 cm H_2_O was used to perform the robotic procedure. VATS segmentectomies were carried out via a 3-port anterior approach according to the Copenhagen technique, as previously described.^10^ Indocyanine green (ICG) was administered intravenously to identify the intersegmental planes during surgery. Lymph node dissection was performed in accordance with the NCCN guidelines for NSCLC.^11^

Simple segmentectomy was defined as segmental resection of the right or left segment 6, or basilar segments in the lower lobes, and upper division (tri-segment) or lingula in the left upper lobe, in which only one linear intersegmental plane is divided. Complex segmentectomy includes all other segmentectomies in which more than one intersegmental plane is divided.^12^

A single drain measuring 24 or 28 Fr was placed at the end of each procedure. Locoregional analgesia was administered via intercostal or paravertebral blocks. Postoperative management was performed according to a predefined departmental protocol regardless of surgical approach.

### Statistical analysis

All statistical analyses were conducted using R (version 4.2.3). Descriptive summaries and group comparisons were performed using the *gtsummary* and *tableone* packages. Competing risks were analysed using the *cmprsk* package to estimate cumulative incidence functions (CIF) for cancer-related and non-cancer-related events, with group comparisons performed using Gray’s test and multivariable Fine–Gray models for covariate adjustment.

Restricted mean survival time (RMST) analysis was conducted with the *survRM2* package, estimating survival up to a specified time point (τ). Inter-group differences in RMST were reported with 95% confidence intervals and *P* values, offering an alternative to proportional hazards assumptions. Learning curve effects were evaluated using cumulative sum (CUSUM) analysis via the *qcc* and *ggplot2* packages. Case order was used as a surrogate for surgical experience and stratified by surgical approach (RATS vs VATS). Inflection points were visually identified to delineate transition phases. Risk-adjusted CUSUM (RA-CUSUM) analyses were further performed using logistic regression models (via stats), adjusting for age, ASA grade, and procedural complexity. Survival associations were assessed using multivariable Cox proportional hazards models (*survival, rms*), with stepwise backward elimination for covariate selection (*P* < .05). Proportional hazards assumptions were evaluated using Schoenfeld residuals (*survminer*). Statistical significance was set at *P* < .05.

## Results

### Patient characteristics

We analysed 144 patients who underwent segmentectomy (RATS: *n* = 86; VATS: *n* = 58). The study population had a mean age of 72 ± 8.3 years, with female predominance (63.9%; *n* = 92). Mean tumour size was 17.2 ± 5.1 mm, with adenocarcinoma being the most common histology (78.5%; *n* = 113). Complex segmentectomies comprised 61.8% (*n* = 89) of procedures. Ten patients received adjuvant therapy (VATS: *n* = 3, 3.4%; RATS: *n* = 7, 8.1%), primarily chemotherapy or chemoradiotherapy. Baseline characteristics were comparable between groups (**Table 1**).

**Table 1.** Patients’ Characteristics

Patients characteristics	VATS	RATS	*P*-value
**Patients (total = 144)**	58	86	
**Age (years)**	72 ± **8**	72 ± **8**	.556
**Female sex**	60.3% (*n* = 35)	66.3% (*n* = 57)	.484
**Smoking status **			
Never	15.5% (*n* = 9)	17.4% (*n* = 15)	.823
Former	60.3% (*n* = 35)	60% (*n* = 49)	.732
Current	24.1% (*n* = 14)	25.6% (*n* = 22)	>.999
**Performance status**			>.999
<2	65.2% (*n* = 38)	66.3% (*n* = 57)	
≥2	34.5% (*n* = 20)	33.7% (*n* = 29)	
**Comorbidities**			
COPD	32.8% (*n* = 19)	34.9% (*n* = 30)	.859
CAD/IHD	8.6% (*n* = 5)	8.1% (*n* = 7)	>.999
CKD	5.2% (*n* = 3)	2.3% (*n* = 2)	.392
DM	10.3% (*n* = 6)	9.3% (*n* = 8)	>.999
TIA/CVA	6.9% (*n* = 4)	7% (*n* = 6)	>.999
**Respiratory function**			
FEV1% predicted (median; IQR)	91.4% [IQR: 30]	86.2% [IQR: 25.1]	.196
DLCO (median; IQR)	78.3% [IQR: 35]	75.8% [IQR: 26.7]	.499
**Segmentectomy type**			
Simple segmentectomy	62.1% (*n* = 36)	61.6% (*n* = 53)	>.999
Complex segmentectomy	37.9% (*n* = 22)	38.4% (*n* = 33)	>.999
**Tumour size (mm)**	17.4 ± **5.2**	17.1 ± **5.1**	.474
**Nodal status**			
pN1	1.7% (*n* = 1)	3.5% (*n* = 3)	.648
pN2	3.4% (*n* = 2)	4.6% (*n* = 4)	>.999
**Tumour histology**			.310
Adenocarcinoma	74.1% (*n* = 43)	81.4% (*n* = 70)	
Squamous cell carcinoma	25.9% (*n* = 15)	18.6% (*n* = 16)	
**Adjuvant therapy**	3.4% (*n* = 3)	8.1% (*n* = 7)	.740

Abbreviations: DLCO, diffusing capacity of the lungs for carbon monoxide; FEV1, forced expiratory volume in 1s.

### Survival associations

Survival associations are shown in **Table 2**. Each additional pack-year of smoking statistically increases risk of death (*P* < .001). Patients with pre-operative lymph node involvement have 3× mortality risk and 4× risk of recurrence (*P* = .048 and *P* = .038, respectively). In patients who experience recurrence, mortality rate is doubled (*P* = .038).

**Table 2.** Cox Proportional Hazards Multivariable Modelling (Stepwise Backward Elimination) for Mortality and Recurrence: Significant Independent Predictors of Survival Across Study Cohort

	Mortality			Recurrence		
Characteristic	HR	95% CI	*P*-value	HR	95% CI	*P*-value
**Pack years**	1.02	1.01-1.04	**<.001**	1.01	1.00-1.03	.11
**Pre-op lymph node involvement**	3.02	1.15-7.94	**.048**	4.02	1.25-12.9	**.038**
**Conversion**			.052			
*No*	—	—		—	—	
*Yes*	15.6	1.59-153				
**Units of blood transfused**	0.55	0.26-1.14	.13			
**Recurrence**	2.32	1.10-4.88	**.038**			
**Age**				0.96	0.91-1.00	.081
**Sex**						.058
*F*	—	—		—	—	
*M*				2.25	0.97-5.19	
**PS ECOG**				1.93	1.06-3.51	**.028**
**Positive N1**				4.30	1.46-12.7	**.031**

Abbreviations: CI, confidence interval; HR, hazard ratio.

The values listed in this table showed significant results: Each additional pack-year of smoking statistically increases risk of death (*P* < .001). Patients with pre-operative lymph node involvement have 3× mortality risk and 4× risk of recurrence (*P* = 048 and *P* = 038, respectively). In patients who experience recurrence, mortality rate is doubled (*P* = 038). Factors that are significantly associated with recurrence include: positive N1 lymph node (*P* = 031) and poorer PS ECOG (*P* = 028).

Factors that are significantly associated with recurrence include: positive N1 lymph node (*P* = .031) and poorer PS ECOG (*P* = .028).

Cox proportional hazards modelling for mortality and recurrence were not significant between surgical groups on multivariable analysis.

#### Schonfield residuals

##### Overall survival

**Figure 1.**
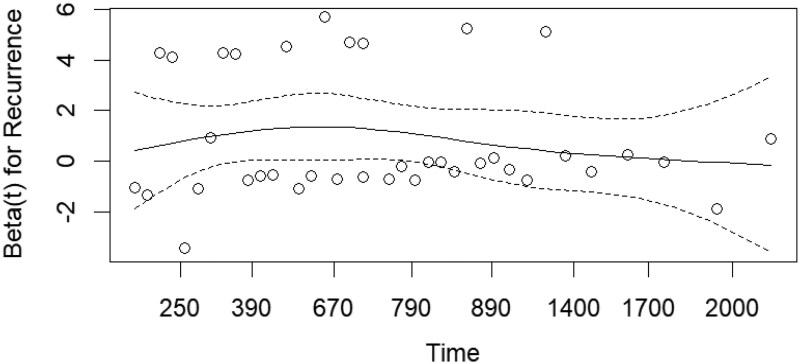
Estimated Time-Varying Coefficient (Solid Line) for Recurrence with 95% Confidence Intervals (Dashed Lines) and Observed Data Points (Circles).

A smoothed estimate of β(*t*)) for recurrence over time shows an approximately flat curve *P* = .59, indicating a stable PH model (**Figure 1**).

##### Disease-free survival

**Figure 2.**
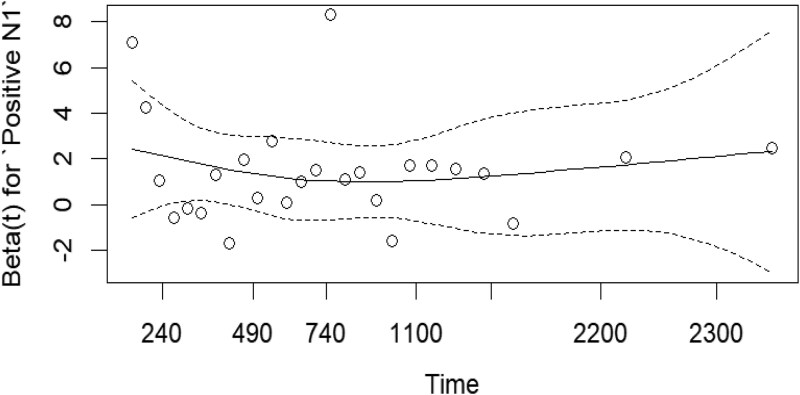
Time-Varying Effect of Positive N1 Status on Outcome, Showing Estimated Coefficient (Solid Line), 95% Confidence Intervals (Dashed Lines), and Observed Data Points (Circles).

An estimated time-varying coefficient β(*t*) for Positive N1 shows an approximately flat solid curve *P* = .433, indicating a stable PH model (**Figure 2**).

### Competing risk analysis for DFS

The cumulative incidence function for relapse over time in the presence of competing events such as mortality showed no difference in recurrence rates by surgical approach (Gray estimate = 1.996, *P* value = .158).

### Overall survival and DFS

Restricted mean survival time analysis (truncated to 780 days) demonstrated no significant difference in average overall or DFS times for either surgical approach (**Table 3**).

**Table 3.** Restricted Mean Survival Time Analysis up to 780 Days Between Surgical Groups

Variable	Approach	RMST (days)	Standard error	95% Confidence interval	*P*-value
Overall survival (days)	VATS	716.4	20.2	676.737-756.043	.367
	RATS	738.8	14.4	710.532-767.117	
Disease-free survival (days)	VATS	735.3	17.4	701.273-769.379	.597
	RATS	746.9	13.2	720.975-772.735	

Abbreviations: RATS, Robotic-assisted thoracic surgery; RMST, restricted mean survival time, VATS, video-assisted thoracic surgery.

### Secondary outcomes

There was no significant difference between the 2 groups in LOS, LOD, complication rate, as well as in operating time and conversion rate, as shown in **Table 4**. The postoperative mortality 90 days after surgery was zero in both groups.

**Table 4.** Secondary Outcomes

Short-term outcomes	VATS	RATS	*P*-value
LOS (median; IQR)	5 [IQR: 5] days	5 [IQR: 5] days	.773
LOD (median; IQR)	2 [IQR: 2.75] days	2 [IQR: 3] days	.089
Complication rate	34.5% (*n* = 20)	34.9% (*n* = 30)	>.999
90-days mortality	0.0% (*n* = 0)	0.0% (*n* = 0)	>.999
Operating time (median; IQR)	120 [IQR: 15] min	115 [IQR: 29] min	.182
Conversion rate	3.4% (*n* = 2)	2.3% (*n* = 2)	>.999
Lymph node stations harvested (median; IQR)			
Overall	5 [IQR: 2]	7 [IQR: 2]	<.001
N1	2 [IQR: 1]	3 [IQR: 1]	<.001
N2	3 [IQR: 1]	4 [IQR: 1]	<.001
Margins free of tumour (median; IQR)	11 [IQR: 15] mm	24.5 [IQR: 18.25] mm	<.001
Nodal upstaging rate	5.2% (*n* = 3)	8.1% (*n* = 7)	.740

Abbreviations: LOS, length of hospital stay; LOD, length of chest drain.

Compared with VATS, RATS was associated with a significantly higher median number of lymph node station harvested overall (5 [IQR: 2] vs 7 [IQR: 2], respectively; *P* < .001), hilar (2 [IQR: 1] vs 3 [IQR: 1], respectively; *P* < .001), and mediastinal (3 [IQR: 1] vs 4 [IQR: 1], respectively; *P* < .001). In addition, RATS was associated with a median margin free of tumour three times wider than VATS (24.5 mm [IQR: 18.25] vs 11 mm [IQR: 15], respectively), and this difference was statistically significant (*P* < .001).

Finally, there was no statistically significant difference in nodal upstaging rate among the groups.

### Learning curve effect

**Table 5** presents a comparison between the steady and learning phases. The steady phase shows a greater number of cases compared to the learning phase. RATS is predominantly performed in the steady phase (71%). The age and sex distributions are almost identical between the two groups. The ECOG score is comparable, though it shows healthier patients in the learning phase (ECOG 0 30% vs 20% in the steady phase). The operative time was slightly shorter in the steady phase (109 vs 115 min).

**Table 5.** Differences Between Learning and Steady Phases

Characteristic	**Learning, *N* = 30** ^a^	**Steady, *N* = 114** ^a^
Case ID	32 ± 28	83 ± 38
Case order	16 ± 9	88 ± 33
Approach		
RATS	5 (17%)	81 (71%)
VATS	25 (83%)	33 (29%)
Sex		
F	19 (63%)	74 (65%)
M	11 (37%)	40 (35%)
Age	72 ± 8	72 ± 8
PS ECOG		
0	9 (30%)	23 (20%)
1	14 (47%)	59 (52%)
2	7 (23%)	32 (28%)
Smoking status		
0	7 (23%)	17 (15%)
1	15 (50%)	79 (69%)
2	8 (27%)	18 (16%)
Complexity	0 (NA%)	0 (NA%)
Unknown	30	114
Date of surgery	February 11, 2017 04:48:00 ± 24660236.2487551	June 21, 2020 09:53:41 ± 33353541.0805925
OR time	115 ± 30	109 ± 36
Conversion		
0	28 (93%)	111 (97%)
1	2 (6.7%)	3 (2.6%)
Reoperation		
0	29 (97%)	112 (98%)
1	1 (3.3%)	2 (1.8%)
In-hospital complications		
0	22 (73%)	72 (63%)
1	8 (27%)	42 (37%)
Mortality		
0	13 (43%)	93 (82%)
1	17 (57%)	21 (18%)
OS (days)	1430 ± 893	1148 ± 473
Recurrence		
0	18 (60%)	100 (88%)
1	12 (40%)	14 (12%)
DFS (days)	1430 ± 893	1148 ± 473
deviation		
–0.1	22 (73%)	72 (63%)
0.9	8 (27%)	42 (37%)
cusum_complication	3 ± 2	20 ± 9
predicted_risk	0.34 ± 0.09	0.35 ± 0.11
ra_cusum	6 ± 3	28 ± 11
conv_risk	0.03 ± 0.05	0.04 ± 0.06
ra_cusum_conversion	365 ± 186	1678 ± 616
expected_time	109.97 ± 1.07	110.02 ± 1.00
cusum_op_time	177 ± 49	389 ± 306

a Mean ± SD; *n* (%); 0 = VATS; 1 = RATS.

Abbreviations: DFS, disease-free survival; OS, overall survival; RATS, Robotic-assisted thoracic surgery; VATS, video-assisted thoracic surgery.

The conversion, reoperation, and in-hospital complication rates were comparable between the learning and steady phases; however, the percentages were higher in the VATS group, as shown in the table. The mortality rate is significantly higher in patients undergoing VATS in the steady phase (82%). Regarding the recurrence rate, it is higher in patients undergoing VATS and mainly in the steady group (88%, vs 60% in the learning group). The OS and DFS are lower in the steady state as compared to the learning state (1148 ± 473 days vs 1430 ± 893 days). All CUSUM values, including complication, RA CUSUM, conversion CUSUM, OR time CUSUM and the others are much higher in the steady group, reflecting cumulative cases over time.

**Figure 3.**
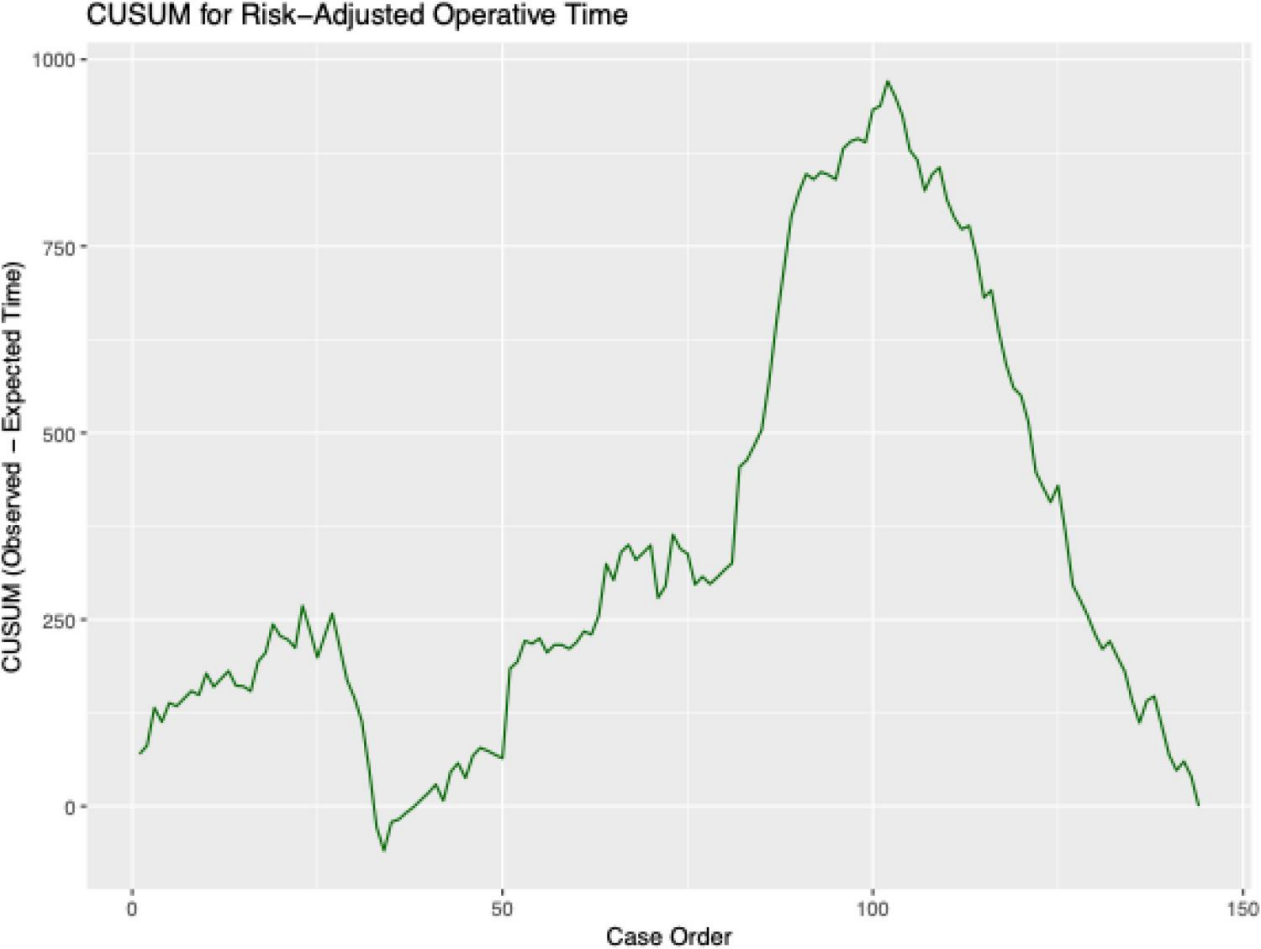
CUSUM Plot for Risk-Adjusted Operative Time Showing Initial Learning Phase with Longer Operative Times (Peak at Case 100) Followed by Progressive Improvement and Stabilization.

Figure of CUSUM for risk-adjusted operative time: (**Figure 3**).

The cumulative sum (CUSUM) curve demonstrates an initial upward rise, indicating operative times greater than expected during the early learning phase. A subsequent decline between cases 25 and 50 reflects early improvements in efficiency.

This inflexion marks the transition from the learning phase to the steady-state phase.

A prolonged rise up to case ∼100 suggests a period of increased case complexity or process adaptation.

Afterwards, the curve declines sharply, indicating sustained reductions in operative time relative to expected values, denoting procedural proficiency.

## Discussion

For early-stage NSCLC, surgical resection is considered the gold standard treatment.

Lobectomy is considered the gold standard resection for early-stage lung cancer, while segmentectomy tends to be employed for sub 2 cm tumours and in cases where pulmonary reserve is poor to begin with. Segmentectomies, however, often require a more thorough and challenging parenchymal dissection.

The VIOLET trial^13^ showed that thoracoscopic minimally invasive surgery is a feasible and effective approach for the surgical treatment of early-stage cancer and is associated with less postoperative pain, fewer complications, and shorter length of hospitalization, without affecting the long-term oncologic outcomes when compared to open surgery. In the past few decades, the robotic approach has been widely used for lung resection surgery because of its advantageous technical features, such as three-dimensional visualization and multi-wristed instruments that allow a more thorough and precise dissection compared to video thoracoscopic surgery. There are some evidences in the literature that RATS might be associated with similar, or even better, short-term outcomes, and comparable long-term survivals compared to VATS.^4^^,^^8^^,^^9^ RAVAL trial’s early results suggest that robotic lobectomy is cost-effective and associated with comparable short-term patient-reported health utility scores when compared with VATS lobectomy.^4^ Kneuertz et al,^9^ in their large propensity-score matched study comparing VATS, RATS and open lobectomy, report a similar recurrence rate between the approaches, (*P *= .9), as well as equivalent 5-year overall (55%, 63%, and 65%, respectively; *P *= .56); however, there is a lack of evidence in current literature on long-term survivals following a segmentectomy comparing the 2 minimally invasive approaches.

In this cohort of patients undergoing segmentectomy by either RATS or VATS, baseline characteristics were largely comparable between groups. RATS was associated with a significantly greater number of lymph node stations harvested—overall, hilar, and mediastinal—and achieved wider tumour-free margins compared with VATS. These findings suggest inherent advantages of the robotic approach, particularly in achieving precise parenchymal dissection and thorough lymphadenectomy. The importance of complete resection is underscored by the complexity of pulmonary lymphatic drainage, which follows both peribronchial and visceral pleural pathways at the segmental level.^14^^,^^15^ Fourdrain et al. demonstrated through cadaveric and surgical specimen studies that intersegmental lymphatic drainage through the visceral pleura occurs in 66% of cases.^16^^,^^17^ This finding has significant implications for sublobar resections, as incomplete resection of lymphatic pathways may increase local recurrence risk and compromise survival. The enhanced precision of robotic segmentectomy may enable more complete removal of the target segment and associated lymphatic drainage pathways, potentially explaining the improved long-term outcomes compared to VATS.

Another factor that has been proven to be linked to better survival by many authors is a more thorough lymphadenectomy.^18–20^ The European Association of Thoracic Surgeons (ESTS) guidelines recommend a systematic nodal dissection in all cases.^19^ The International Association for the Study of Lung Cancer (IASLC) defines a systematic nodal dissection as the excision of at least three N2 stations, including the subcarinal station, and three N1 stations.^20^ Furthermore, in the upcoming 9th edition of the lung TNM, 2 additional subcategories have been added to the N2 category: the subcategory N2a, which denotes metastasis limited to a single ipsilateral mediastinal or subcarinal station, and the N2b subcategory, which indicates metastases in multiple mediastinal or subcarinal stations.^21^ This important subdivision was made because survival analyses demonstrated a clear and consistent prognostic difference between single and multiple N2 station involvement in both clinical and pathological stages. These results underscore the importance of an adequate systematic lymphadenectomy, which is essential in lung cancer surgery to obtain a correct postoperative staging of the disease, so as to identify patients who need adjuvant therapies. In addition, an adequate lymphadenectomy is fundamental to ensure a complete resection, removing possible nodal metastatic and micrometastatic disease.^22^^,^^23^ A meta-analysis by Jeong et al.^23^ showed that nodal micrometastases were detected in 25.3% of 2026 NSCLC cases without nodal involvement in histologic examination, and that the presence of nodal micrometastases was significantly related to a higher recurrence rate and worse survival.

The robotic approach has been associated in various studies with a higher lymph node yield.^8^^,^^24^^,^^25^ From the partial results of the ongoing clinical trial made by Patel et al,^4^ RATS demonstrated to be associated with a higher number of lymph nodes harvested compared with VATS (10 [IQR: 8-13] vs 8 [IQR: 5-10]; *P* = .0003). Similarly, in our series, RATS was associated with a higher median number of lymph node station harvested overall (7 [IQR: 2] vs 5 [IQR: 2], respectively; *P *< 0.001), mediastinal (4 [IQR: 1] vs 3 [IQR: 1], respectively; *P *< 0.001) and hilar (3 [IQR: 1] vs 2 [IQR: 1], respectively; *P *< .001) compared with VATS.

In addition, various studies show that a wider bronchial margin might be associated with improved survival.^26^^,^^27^ Mohiuddin et al.,^26^ in their large cohort study, demonstrated that patients who underwent a wedge resection with a 15 mm margin distance had a risk of recurrence reduced by 59% compared to patients with a 5-mm margin distance. A retrospective study by Wolf et al.^27^ illustrated how increased margin distance was independently associated with lower recurrence risk (odds ratio [OR]: 0.90; 95% CI, 0.83-0.98) and improved OS (HR: 0.94; 95% CI, 0.90-0.98) for each 1 mm increase. In our study, RATS was associated with a median resection margin free of tumour of 24.5 mm [IQR: 18.25] compared to 11 mm [IQR: 15] associated with the VATS approach (*P* < .001); furthermore, in our series, the majority of the recurrences in the VATS group were local (6 over 10 patients with a recurrence), and the local recurrence rate following a VATS segmentectomy was three times higher than after RATS segmentectomy (10.3% vs 3.5%, respectively) even though this difference did not result statistically significant (*P* = .158).

The improved long-term survival and lower recurrence rates, particularly local recurrences, observed with RATS in our study may be attributed to the enhanced precision of robotic dissection in both the intersegmental plane and lymph node harvesting. However, we acknowledge potential selection biases and unmeasured confounders that may have favoured the RATS group independently of surgical technique.

Regarding short term outcomes, in numerous studies, the robotic approach was associated with similar or even improved short-term outcomes, both following a lobectomy^4^^,^^5^ and a segmentectomy.^6^^,^^7^ Zhang et al.^6^ in their multi-institutional propensity matched analysis, showed that there were no statistically significant difference between RATS and VATS segmentectomy in terms of conversion rate (1 vs 3; *P* = .624), operative time (147.91 ± 52.42 .vs 149.23 ± 49.66 min; *P* = 0.773), rates of overall complications (17.9% vs 14.8%; *P* = .340), and length of stay (4 days [IQR, 3-5 days] vs 4 days [IQR, 3-5 days]; *P* = .417). Our results are in line with the aforementioned findings, with no statistically significant difference in LOS, complication rate, operating time, and conversion rate, suggesting that RATS is at least as feasible and safe as the video-assisted approach to perform a segmentectomy.

### Strengths and limitations of the study

A key strength of our study is its robust follow-up data, with a median duration of 33 months and no patients lost to follow-up, increasing the reliability of our survival and oncological outcomes analysis.

However, several limitations warrant consideration. The retrospective design introduces potential selection bias, and the lack of randomization may result in both measured and unmeasured confounding factors. Additionally, surveillance duration differed between groups (VATS: 45 months; RATS: 26 months), reflecting our centre’s more recent adoption of the robotic approach in 2017. Furthermore, while the VATS cohort represents mature experience (>5 years), the RATS group includes our learning curve period. These limitations highlight the need for large-scale, multicentre randomized trials to validate our findings, and longer follow-up is needed to confirm the durability of RATS outcomes.

## Conclusion

In our series, robotic segmentectomy was associated with significantly improved overall survival, DFS, and lower recurrence rates compared to the video-assisted approach. RATS enabled more extensive lymphadenectomy and wider resection margins while maintaining comparable perioperative outcomes to VATS, suggesting that the enhanced technical precision of the robotic platform may translate into improved oncological outcomes. While our findings support the adoption of robotic techniques for anatomical segmentectomies, further studies are needed to validate these results.

## Data Availability

The data presented in this study are available on request from the corresponding author. The data are not publicly available.
